# Barriers and associated factors for adequate antenatal care among Afghan women in Iran; findings from a community-based survey

**DOI:** 10.1186/s12884-020-03121-5

**Published:** 2020-07-28

**Authors:** Omid Dadras, Fateme Dadras, Ziba Taghizade, Seyedahmad Seyedalinaghi, Masako Ono-Kihara, Masahiro Kihara, Takeo Nakayama

**Affiliations:** 1 Department of Health Informatics, Graduate School of Medicine, Kyoto University, Kyoto, Japan; 2 Department of Obstetrics and Gynecology, Tehran University of Medical Science, Tehran, Iran; 3 Nursing and Midwifery Care Research Center, Tehran University of Medical Science, Tehran, Iran; 4 Iranian Research Center for HIV/AIDS, Iranian Institute for Reduction of High-Risk Behaviors, Tehran University of Medical Sciences, Tehran, Iran; 5 Global Health Interdisciplinary Unit, Center for Promotion of Interdisciplinary Education and Research, Kyoto University, Kyoto, Japan

**Keywords:** Antenatal care, Access, Refugee and immigrant, Afghan women, Iran

## Abstract

**Background:**

Almost a third of Afghan women living in Iran are at childbearing age. Antenatal care (ANC) is an inextricable part of healthy pregnancy and could prevent the adverse birth outcomes. Almost 97% of Iranian expectant women are receiving adequate ANC (4 or more visits). However, the situation for pregnant Afghan women is unclear. Some studies indicated low access to ANC among Afghan women. In the present study, we aimed to explore the sociodemographic factors and potential barriers associated with adequate ANC among Afghan women in Iran.

**Methods:**

A cross sectional study was conducted between June 2019 and August 2019. Using time location sampling (TLS), we recruited 424 Afghan women aged 18–45 years old at three health centers in south region of Tehran. The data were collected on sociodemographic characteristics and the reported reasons for inadequate ANC using a questionnaire and analyzed applying bivariate, and multivariate analyses. Factor analysis was performed to reduce the number of potential reasons for inadequate ANC in order to improve the precision of regression analysis.

**Results:**

Almost a third of Afghan women in this study had adequate ANC (≥ 8 visits). The women in older age group, those with higher education and family income, women with longer length of stay, those of legal status were more likely to have adequate ANC. In multivariate analysis, the poor knowledge and attitude toward ANC (AOR = 0.06; 95% CI [0.03–0.15]), the poor quality of services (AOR = 0.17 95% CI [0.07–0.41]); and to some extent, the difficulties in access (AOR = 0.33; 95% CI [0.11–1.00]) were the main obstacles toward adequate ANC among the study population.

**Conclusion:**

Our study emphasized the important role of the personal knowledge and attitude toward ANC with adequate antenatal care among Afghan women in Iran. This could be addressed by well-oriented interventions and health education for Afghan women. The collaboration between central government with international agencies should be directed toward enhancing the social support, promoting the awareness and knowledge, and expanding the safety net services to improve the access and quality care among Afghan women in Iran.

## Background

The maternity care is of great importance in normal growth and development of baby in prenatal period. A successful fetal life and safe delivery not only ensure the health of newborns but also has a grave impact on their adult life. Therefore, antenatal care is an inextricable part of maternal and child health [1, 2]. Perinatal health depends on a variety of factors and could differ to a great extent in different populations [3]. Most of the child problems such as congenital abnormalities, prematurity, and abnormalities in fetal growth could be prevented by early diagnosis in antenatal period. Therefore, adequate antenatal care (ANC) is essential to ensure the child and maternal health [4]. Studies have shown that the perinatal mortality and morbidity could be three times higher in some underprivileged areas and populations whose access to ANC is inadequate [5].

The latest statistics indicate 97% of pregnant Iranian women attend the WHO recommended ANC during their pregnancy [6]. Furthermore, the youth literacy rate for females (98%), institutional birth rate (95%), total fertility rate (1.9%), and MMR (25/100,000 live births) are all indicative of Iran’s achievements in improving women’s health [7]. Iran is one of the successful countries in MENA region that achieved the MDGs. By year 2015, Iran reduced the MMR by 75%, reaching to the highest reduction compared to neighboring countries [8]. However, the population specific data especially among immigrants and refugees are missing in national statistics. This creates a huge gap in understanding and addressing the current health situation and issues of this vulnerable population.

Immigrants and refugees are among the most vulnerable populations in host country; the violence, discrimination, and deprivation of civil rights in host country could endanger their health, particularly the vulnerable groups such as pregnant mothers and children [9]. Inadequate access to appropriate antenatal care contributes to a substantial amount of adverse birth outcomes [5]. Iran has been a popular destination for Afghan refugees and immigrants since the Soviet war in 1979, with an estimated 3 million Afghans refugees and immigrants currently living in the country. About half of them are not registered and considered as illegal immigrants [10, 11]. The majority of them are young and almost a third are young women at the child bearing age [12].

Since 2016, all legal Afghans in Iran became eligible to apply for Public Health Insurance (PHI) [9]. The insured person could benefit from the low-cost and affordable primary health care services such as primary health check-up, routine screening, vaccination, antenatal care, and basic laboratory tests provided at government facilities. However, PHI does not cover the cost for some services such as diagnostic or screening sonography at the secondary or tertiary levels especially in private facilities. This contributes to the low uptake of this insurance by Afghans. In a preliminary qualitative study, we found that it is either due to the unawareness or dissatisfaction of PHI coverage [13]. Unfortunately, there is lack of further knowledge in this regard and further studies are required. The important fact is that the lack of health insurance could reduce the access to health system for Afghans in Iran. It is more critical for the women of childbearing ages whose health, especially during the pregnancy, is critical to prevent the adverse birth outcomes [14]. Therefore, building capacity to facilitate the access to health system for this vulnerable group and addressing their maternity and reproductive needs are necessary.

Beside the lack of health insurance, several obstacles could reduce the access to quality and adequate maternity care among Afghan women in Iran. In previous studies, navigating ANC services, mothers’ perception of quality of care, socioeconomic status, language, cultural, religious concern were some the most frequent reported reasons hindering the adequate access to ANC among immigrants and refugees [15]. However, the diversity in context and population-specific factors could contribute to variety in issues and concerns in accessing health system in different host countries. Therefore, a comprehensive understanding of Afghan women’s demographic and socio-cultural characteristics, their current health situation as well as the knowledge of potential barriers toward adequate ANC are essential to formulate appropriate interventions to improve the access and utilization of ANC among Afghan women in Iran. Thus, in present study, we aimed to fulfil these objectives by conducting a community-based survey among pregnant Afghan women.

In a preliminary qualitative research in 2019, we explored the potential barriers and concerns of Afghan women toward adequate antenatal care (ANC) in Iran [13]. We interviewed 30 Afghan women living in Tehran and the results have been used to create the platform and develop and questionnaire and conduct the present survey in order to estimate the prevalence of associated factors and potential barriers toward adequate maternity care among Afghan women in Iran. In present study, we conducted a survey including 424 Afghan women of both legal and illegal backgrounds to estimate the prevalence of identified barriers and examine the association of sociodemographic factors and perceived barriers with the adequate utilization of ANC.

## Methods

### Study setting

A cross sectional study was conducted among Afghan women from June 2019 to August 2019. Based on the consultation with the experts at Tehran University of Medical Sciences and Iran Ministry of Health, the south region of Tehran where the highest number of Afghan nationals are living, was selected as the study setting. This region is named Share-Rey and located in south-east part of Tehran province. There are 15 urban community health centers governed by regional health center.

### Sample size

Based on the literature review [16], with the conservative assumption of access to antenatal care not to be less than 50%, confidence level of 95%, a relative precision of 5% points on each side, and a response rate of 90%, the sample size was estimated at 424. However, we could conduct 427 face to face interviews with 100% response rate. There was no drop out or withdrawal during the study.

### Sampling method

Afghan population, particularly the illegal ones, are hard to reach in Iran; therefore, we employed a time –location sampling (TLS) approach to recruit the participants in the present study. TLS is a method for reaching individuals in places and at times where they congregate rather than where they live. It is a method of sampling used to collect data from hard-to-reach populations, such as men who have sex with men (MSM), injection drug users, artists, high-risk youth, refugees and migrants, and other populations that form social networks who can be found at identifiable locations [17]. Following this TLS principles; first, we identified the common places and times that Afghan females visit to receive maternity care. During a primary survey, it appeared that the urban community health centers are the most popular places for maternity care among Afghan women and they usually visit these centers in the morning times. Therefore, all the community health centers in south region of Tehran (15 centers) were identified and three centers with the highest number of Afghan visitors were selected as the study sites. We visited these centers in the morning time during the working days of four consecutive weeks in July, 2019. A sample of 424 Afghan women, who met the eligibility criteria and consent to participate, were recruited proportionately to the size of Afghan population visiting the corresponding community health center.

### Inclusion and exclusion criteria

The eligibility criteria were being an Afghan national, aged 18–49 years, given birth or being pregnant during the year before interview, and lived in Iran at least a year and during the time of pregnancy. We excluded the women with any mental or physical disabilities that affect their access to maternity care, those who visited or stayed in Afghanistan during their pregnancy, those who suffer from infertility, and those who did not consent to participate.

### Instrument and variables

The questionnaire (supplementary 1) was developed based on the findings from a preliminary qualitative research, a comprehensive literature review, and consulting the experts at Kyoto University and Tehran University of Medical Sciences. The questionnaire collected the data on participants’ sociodemographic characteristics including the age, education, employment, family income (< 4million vs ≥4million-Iranian Toman), legal stratus (legal vs illegal), length of stay (< 5 years vs ≥5 years), parity, husband employment, and education. It also contained 17 questions asking about the barriers to ANC identified in the preliminary qualitative study. The answers were marked as yes/no. In addition, data on the number of ANC (< 4, 4–8, ≥ 8), usual place of visit for ANC (community health center, government hospital, private sector) were also collected. The optimal number of ANC is ≥8 visits during pregnancy based on the WHO recommendation (2016). Therefore; in our study, the cut-off for adequate ANC is 8 antenatal visits [18].

### Data collection

A pilot study, including 12 participants was performed to test whether the questionnaire was comprehensible and appropriate, and that the questions were well defined, clearly understood and presented in a consistent manner. Patient information statement and consent forms were also tested for comprehension. The main interview took place after the pilot study. The interviewers were Afghan graduate midwifery students in Tehran University of Medical Sciences. The participants were approached by the interviewers during the visit of community health center. A brief introduction of study and its objectives was provided for each participant prior to the interview. Given a verbal consent, the woman was entered the study and a written consent was obtained before the interview. Data were collected in the questionnaire through face to face interview. Interviews were conducted in Persian language and each lasted approximately 15 min. At the end of interview, the women’s concern regarding healthy pregnancy, child health, and nutrition were addressed by the interviewers.

### Data analysis

The descriptive statistics were employed to describe the distribution of sociodemographic characteristics, the number of ANC (Table 1), and the prevalence of potential barriers and the reasons for inadequate ANC (Table 3) among Afghan women. To examine the relationship between sociodemographic factors (Table 2) and reported reasons (Table 3) with inadequate ANC (< 8 visits), chi-square test was employed and the crude odds ratios (COR) and 95% confidence intervals (95%CI) were reported (Tables 2 and 3). The exploratory factor analysis was employed to explore the potential correlation between reported reasons for inadequate ANC and extract the underlying component factors grouping the correlated variables (eigenvalues > 1). This helped us to reduce the number of variables and thus to increase the power of regression analysis. Four component factors were emerged after varimax rotation; including poor knowledge/attitude toward ANC, difficulties in access, poor quality of services, and sociocultural/legal issues (Table 4). The association between these component factors and adequate ANC was also examined (Table 4). Eventually, multivariate logistic regression analysis was performed, including the sociodemographic variables and component factors in equation, to explore the relationship between the component factors with inadequate ANC after adjusting for sociodemographic variables (Table 5). The variables with *P*-value < 0.25 in bivariate analysis, were included in regression equation following the instructions of Hosmer and Lemeshow [19]. In a step-wise manner, retaining the significant factors in equation, the best regression model was constructed using the likelihood-ratio test for goodness of fit. The results represented as adjusted odds ratio (AOR). The STATA software version 14 was used for data analysis. P-value less than 0.05 was determined as significant statistical level.

**Table 1:** The distribution of sociodemographic characteristics of participants

	*n (%)*
Age group (year)	
18–24	131 (30.9)
25–34	188 (44.3)
35–44	105 (24.8)
Education	
Illiterate	160 (37.7)
Literate	264 (62.3)
Employment	
Employed	216 (50.9)
Unemployed	208 (49.1)
Parity	
1	80 (18.9)
2–4	240 (56.6)
≥ 5	104 (24.5)
Husband education	
Illiterate	159 (37.5)
Literate	265 (62.5)
Husband employment	
Employed	416 (98.1)
Unemployed	8 (1.9)
Family income (Iranian Toman)	
< 4 million	224 (52.8)
≥ 4 million	200 (47.2)
Length of stay in Iran	
< 5 years	192 (45.3)
≥ 5 year	232 (54.7)
Legal status	
Illegal	160 (37.7)
Legal	264 (62.3)
Insurance *	
No insurance	144 (54.5)
Had insurance	120 (45.5)
The number of ANC	
< 4	64 (15.1)
4–8	208 (49.1)
≥ 8	152 (35.8)
Total	424 (100)

* Only included the legal immigrant (*n* = 264)

**Table 2:** The association between the sociodemographic factors and adequate ANC (≥ 8 visits) in Afghan women

		Number of ANC			*p-value*
		< 8 visits (%)	≥ 8 visits (%)	Crude OR (95% CI) ^a^	
Age group (year)	18–24	70 (25.7)	61 (40.1)	Ref	
	25–34	137 (50.4)	51 (33.6)	0.43 (0.27–0.68)	
	35–45	65 (23.9)	40 (26.3)	0.71 (0.42–1.19)	0.002
Education	Illiterate	120 (44.1)	40 (26.3)	Ref	
	Literate	152 (55.9)	112(73.7)	2.21 (1.43–3.41)	0.001
Employment	Unemployed	128 (47.1)	88 (57.9)	Ref	
	Employed	144 (52.9)	64 (42.1)	0.65 (0.43–0.96)	0.032
Parity	1	48 (17.6)	32 (21.1)	Ref	
	2–4	152 (55.9)	88 (57.9)	0.87 (0.52–1.46)	
	≥ 5	72 (26.5)	32(21.1)	0.67 (0.36–1.23)	0.399
Husband education	Illiterate	128 (47.1)	31 (20.4)	Ref	
	Literate	144 (52.9)	121 (79.6)	3.47 (2.19–5.50)	0.001
Husband employment	Employed	266 (97.8)	150 (98.7)	Ref	
	Unemployed	6 (2.2)	2 (1.3)	0.59 (0.12–2.97)	0.518
Family income (Iranian Toman)	< 4 million	184 (67.6)	40 (26.3)	Ref	
	≥ 4 million	88 (32.4)	112 (73.7)	5.85 (3.77–9.10)	0.001
Length of stay in Iran	≤ 5 years	152 (55.9)	40 (26.3)	Ref	
	> 5 year	120 (44.1)	112 (73.7)	3.55 (2.30–5.47)	0.001
Legal status	Illegal	136 (50.0)	24 (15.8)	Ref	
	Legal	136 (50.0)	128 (84.2)	5.33 (3.25–8.76)	0.001
Insurance *	No insurance	112 (82.4)	32 (25.0)	Ref	
	Has insurance	24 (17.6)	96 (75.0)	14.00 (7.71–25.39)	0.001*

* Only included the legal immigrant (*n* = 264)

^a^ The odds ratio (OR) and 95% confidence interval (95%CI) for adequate access to ANC (≥ 8 visits)

**Table 3:** The distribution of potential reasons and their association with adequate ANC (≥ 8 visits) in Afghan women

	Number of ANC		*p*-value
	< 8 visits (%)	≥ 8 visits (%)	
I was healthy	136 (50.0)	64 (42.1)	0.118
I think it is unnecessary	176 (64.7)	48 (31.6)	0.001
Expenses were unaffordable	192 (70.6)	72 (47.4)	0.001
Clinic was too far	184 (67.6)	48 (31.6)	0.001
Family members disapproved^a^	16 (5.9)	8 (5.3)	0.791
Poor transportation	216 (79.4)	72 (47.4)	0.001
Scare of / being discriminated or poorly treated	168 (61.8)	64 (42.1)	0.001
No one to accompany me	120 (44.1)	24 (15.8)	0.001
The services were not good	56 (20.6)	8 (5.3)	0.001
Long waiting time	96 (35.3)	24 (15.8)	0.001
Religious and cultural reasons	144 (52.9)	40 (26.3)	0.001
Better service at home^a^	8 (2.9)	0	0.055
Fear of arrest/deportation	120 (44.1)	24 (15.8)	0.001
Total	272	152	

^a^ These variables were not included in the factor analyses

**Table 4:** Factors emerged in factor analysis and their association with adequate ANC (≥ 8 visits) in Afghan women

	Number of ANC			
	< 8 visits (%)	≥ 8 visits (%)	Crude OR (95% CI)	*p*-value
Poor knowledge/attitude toward ANC^a^	192 (70.6)	72 (47.4)	0.37 (0.25–0.57)	0.001
Difficulties in access^b^	240 (88.2)	104 (68.4)	0.29 (0.17–0.48)	0.001
Poor quality of services^c^	112 (41.2)	32 (21.1)	0.38 (0.24–0.60)	0.001
Sociocultural/legal issues^d^	200 (73.5)	88 (57.9)	0.50 (0.33–0.75)	0.001
Total	272	152		

^a^ composed of: “I was healthy”, “I think it is unnecessary”

^b^ composed of: “Expenses were unaffordable”, “Clinic was too far”, “Poor transportation”

^c^ composed of: “The services were not good”, “Long waiting time”

^d^ composed of: “Scare of / being discriminated or poorly treated”, “Religious and cultural reasons”, “Fear of arrest/deportation”, “No one to accompany me”

**Table 5:** Adjusted Odds ratios (AOR) and 95% Confidence Interval (95% CI) for the effect of sociodemographic factors and potential barriers on the access to ANC (≥8) among Afghan women

		AOR	95% CI	*p*-value
Age group (year)	18–24	Ref		
	25–34	0.24	0.11–0.52	0.001
	35–44	11.61	2.80–48.20	0.001
Education	Illiterate	Ref		
	Literate	8.65	2.48–30.11	0.001
Employment	Unemployed	Ref		
	Employed	0.54	0.18–1.59	0.264
Husband education*	Illiterate	Ref		
	Literate	4.67	1.91–11.40	0.001
Length of stay in Iran (years)	≤ 5	Ref		
	> 5	3.29	1.43–7.61	0.005
Family income (Iranian Toman)	< 4 million	Ref		
	≥ 4 million	8.59	3.45–21.36	0.001
Legal status	Illegal	Ref		
	Legal	7.07	2.28–21.96	0.001
Poor knowledge/attitude toward ANC		0.06	0.03–0.15	0.001
Difficulties in access		0.33	0.11–1.00	0.050
Poor quality of services		0.17	0.07–0.41	0.001
Sociocultural/legal issues		0.46	0.19–1.10	0.080

## Results

### Sociodemographic characteristics

A total number of 424 Afghan women aged 18–45 years old enrolled in this study. The majority of them (44.3%) were in 25–34 age group and had no education (37.7%). Almost half of them had a job. More than half of them (56.6%) had 2–4 pregnancy experiences. In term of the husband education and employment, 62.5% of them were literate and almost all of them (98.1%) were employed. The family income was more than 4 million Toman (11,500 Iranian Toman = 1 USD) in about half of the households. More than 60% of participants were legal immigrants. Only 120 participants (45.5%) of legal immigrants had health insurance (Table 1).

### Access to the ANC

According to the new WHO recommendation (2016) for adequate ANC, almost 36% of Afghan women received adequate ANC (≥ 8) during their pregnancy. However, based on previous WHO recommendation (2002) for adequate ANC (4 or more visits), about 85% of participants had adequate ANC.

### The sociodemographic determinants of adequate ANC

Tables 2 and 5 illustrate the results of bivariate and multivariate analyses for the association of sociodemographic factors with adequate ANC. It appeared that the oldest age group (35–45 years) are less likely to have adequate ANC (at least 8 visits) (26.3%) compared to the younger age groups; however, in multivariate analysis, after adjusting for other sociodemographic variables and component factors, the odds of having adequate ANC in this group became almost 11 times the youngest age group (18–24 years). The higher education level was significantly associated with adequate ANC in bivariate analysis. Likewise, in multivariate analysis, the literate women were more likely to have adequate ANC (AOR = 8.65; 95% CI [2.48–30.11]). It appeared that having a job reduce the likelihood of having adequate ANC in Afghan women (COR = 0.65; 95% CI [0.43–0.96]). However, it became insignificant after adjusting for other variables in regression analysis (AOR = 0.54; 95% CI [0.18–1.59]). There was no significant association between the number of pregnancies and having adequate ANC in bivariate analysis (*p* = 0.399). Therefore, we did not include it in final regression equation. The higher education of husband was significantly associated with adequate ANC in both bivariate and multivariate analyses (AOR = 4.67; 95% CI [1.91–11.40]). There was no significant association between the husband employment and having adequate ANC in bivariate analysis (*p* = 0.518). Having more than 4 million Toman family income was significantly associated with adequate ANC (AOR = 5.85; 95% CI [3.77–9.10]), even after adjustment for other variables in multivariate analysis, the odds of having more than 8 ANC visits was almost 8.59 times higher in women of 4 million or higher family income. It appeared that the access to adequate ANC in settled Afghan refugees and immigrants (with more than 5 years in Iran) is significantly higher than that of the recent ones (5 years or less in Iran) in both bivariate (COR = 3.55; 95% CI [2.30–5.47]) and multivariate analysis (AOR = 3.29; 95% CI [1.43–7.61]). In both bivariate and multivariate analysis, the legal immigrants were more likely to have adequate ANC (AOR = 7.07; 95% CI [2.28–21.96]).

In Iran, only legal immigrants are eligible for having health insurance; thus, this variable was only examined among legal immigrants (Tables 1, 2). The results showed that the odds of having adequate ANC is 14 times higher in insured Afghan women (COR = 14.00; 95% CI [7.71–25.39]).

### The reasons for inadequate ANC

The component factor “poor personal knowledge and attitude toward ANC” was negatively associated with the adequate ANC among Afghan women (COR = 0.37; 95% CI [0.25–0.57]). Likewise, in multivariate analysis, after adjustment for other variables in equation, this relation remained significant (AOR = 0.06; 95% CI [0.03–0.15]).

The hardships in seeking ANC such as unaffordable expenses, far distance, and poor transportation were more prevalent among those with inadequate ANC (70.6, 67.7, and 79.4% respectively). Although in bivariate analysis; the component factor “difficulties in access” was associated with inadequate ANC (COR = 0.29; 95% CI [0.17–0.48]), adjustment for other variables in multivariate analysis, diluted this association (AOR = 0.33; 95% CI [0.11–1.00]).

Poor quality of services appeared to be a significant obstacle in having adequate ANC in both bivariate (COR = 0.38; 95% CI [0.24–0.60]) and multivariate analyses (AOR = 0.17 95% CI [0.07–0.41]).

The sociocultural and legal issues such the fear of being discriminated or poorly treated, religious and cultural concerns, and fear of deportation or arrest were respectively reported among 61.8, 52.9, and 44.1% of those with inadequate ANC. Accordingly, these issues were negatively associated with adequate ANC in bivariate analysis (COR = 0.50; 95% CI [0.33–0.75]). However, adjusting for other variables in multivariate analysis, reduced the cumulative impact of these issues on adequate ANC among Afghan women (AOR = 0.46; 95% CI [0.19–1.10]).

## Discussion

The factors influenced the utilization and access to ANC among refugees and immigrants could be diverse in different host countries [20]. In present study, we evaluated the prevalence and the impact of sociodemographic factors and potential obstacles on the adequate utilization of ANC among Afghan women in Iran. Following a preliminary qualitative research and comprehensive literature review the potential obstacles and concerns of Afghan women in seeking maternity care in Iran were conceptualized to develop the questionnaire of present study. The results indicated that almost a third of Afghan women in this study, had adequate access to ANC (8 or more ANC visits). The Afghan women from the older age group, those with higher education and family income, women with longer length of stay, and those of legal status were more likely to have adequate ANC. The husband education has also a positive association with adequate ANC. Furthermore, owning health insurance proved to be an important determinant of adequate ANC among legal immigrants. In final analysis, only the poor knowledge and attitude toward ANC; poor quality of services; and to some extent, the difficulties in access were the main obstacles in having adequate ANC among the study population.

The education and socioeconomic status are some the most important determinants of access to healthcare [21]. A number of surveys, in both developing and developed countries, have shown that the educated individuals from higher socioeconomic classes are more likely to have access to quality and adequate healthcare [11, 22]. Similar findings have been reported among immigrants and refugees [23–25]. In our study, we found that Afghan women with higher education and family income, regardless of their legal status, are more likely to have adequate ANC. Higher education has been linked to higher chance of finding a job, higher income, and better living condition [21]. Furthermore, it has been shown that the educated mother are more willing to engage in their child health and better recognize the importance of ANC [26]. Clearly, the husband education has the similar impact on the utilization of reproductive and maternity cares. Evidence indicate that men with higher education are more likely to contribute to the reproductive needs of their partner [24, 27].

Another important determinant of maternal and child health is mother’s employment [28, 29]. Previous studies indicated that the employed mothers are more likely to have adequate ANC [29]. In our study, however, we observed that unemployed mothers are more likely to have adequate ANC. The less flexible schedule and autonomy of employed mother could contribute to the inadequate ANC during the pregnancy among Afghan women. Furthermore, the lower chance in securing a decent job, due to the refugee status, often push the Afghan women toward laborious jobs with long working hours and no maternity leave. Moreover, often due to the financial struggles, they cannot afford to quit their job, especially illegal immigrants [30] and this could substantially reduce their autonomy and access to adequate ANC. Therefore, developing and implementing appropriate labor market laws should be the priority of Iranian government to support the maternity leave for refugees and immigrants is the country.

It has been shown that the recent refugees and immigrants (less than 5 years of arrival) have less access to ANC compared to settled ones (more than 5 years of arrival) [24]. Our results similarly indicated that the Afghan women living in Iran more than 5 years are more likely to have adequate ANC. This has been attributed to the higher access to health system achieved through the familiarization, acculturation, and accustomization of settled population with the host country’s health system, culture, and environment [31].

The legal status has been recognized as an important determinant of access to the health system in host country [14, 32]; Several studies have shown that the illegal status of undocumented immigrant could reduce their access to ANC and increase the adverse birth outcomes [20, 33, 34]. Similarly, we observed lower ANC among illegal Afghan women, even after adjustment for other variables. One issue is their ineligibility for health insurance. Currently, all the registered Afghan refugees and immigrants could benefit from public health insurance (PHI) in Iran; however, the situation for illegal Afghans in unclear. In addition, PHI only reimburses for some of ANC services at the primary healthcare level; and the secondary and tertiary prenatal services which are usually expensive are not covered [9]. We found that the insured Afghan women are more likely to have adequate ANC among legal immigrants. Therefore, first, measures to improve the coverage of current insurance scheme and second, laws for universal health coverage of all Afghan refugees and immigrants, at least for the expectant mothers, should be passes by the national government to ensure the maternal and child health in Afghan refugees and immigrants in Iran.

Poor knowledge and attitude toward ANC were important reasons for inadequate ANC among Afghan women in this study. The statement “I was healthy” reflected the Afghan women’s lack of knowledge in term of the ANC importance for a healthy pregnancy. It was reported by almost half of the participates with inadequate ANC. Furthermore, the poor attitude toward ANC (I think it is unnecessary) was observed by almost two thirds of the participants. Previous studies emphasized the importance of well-oriented education programs for enhancing the reproductive and maternal health among immigrants and refugees in host country [35, 36]. Future policies and interventions should be directed toward enhancing the knowledge related to the reproductive health and maternity care among Afghan women in Iran.

The poor quality of health care such as long waiting time and poor quality of antenatal services, was one the most important associated factor with inadequate ANC in present study. This finding was in line with the evidence from other developing countries. The weak infrastructure of health system and inadequate number of health professionals in some remote areas; particularly in government sector, reported to be the main reasons for such issues in most developing countries [37, 38]. Likewise; in Iran, these issues still exists in some remote areas. Although in big cities such as Tehran, these issues are shown not to be problematic anymore [39], our study indicated that these issues still exist in some marginalized communities like Afghans even in big cities like Tehran. They are even more evident in government sector such as community health centers and government hospitals [39]; where not only receive the highest number of Afghan visitors but also many Iranian citizen who are more vulnerable due to their low socioeconomic status. Therefore, the government should direct adequate resources toward building capacity in these underprivileged areas in order to promote the quality services.

Religious and cultural concerns have been reported to be potential obstacles in utilization of ANC and accessing quality ANC services in some strict Islamic states such as Afghanistan [40, 41]. The gender-sensitive issues such as contact restriction between male provider and female patient are some of the reported religious and cultural barriers toward adequate ANC among Afghans. This reduces women access to maternity care; specially in countries of west culture in which these issues are often ignored by health providers [41–43]. However, in our study, due to the dominant Islamic culture of Iranian society in which the contact of a female patient and a male provider is also restricted, these issues appeared not to be problematic. In addition, none of the participants complained about unavailability of a female provider.

Fear of deportation or arrest also appeared to be an important barrier to visit health facilities among illegal Afghan women in our study. We observed this issue in 44% of those with inadequate ANC. Therefore, we suggest expanding the safety net services by collaboration between international and national agencies for refugees support to ensure the adequate access to ANC in this vulnerable group. The fear of being poorly treated or discriminated was also a reason among approximately 60% of those with inadequate ANC in this study. Evidence has shown that the perceived discrimination; feeling of abandonment, and isolation among immigrants and refugees could affect the individual’s perceived quality of life and satisfaction in host society [44, 45]. Similar experiences were reported among Afghan people living in Iran [46]. The intercultural incompetency of health personnel has been reported to be the main reason for such issues. The language barrier could also cause such bitter experiences [47, 48]; however, in Iran, almost all Afghans could fluently speak and communicate in Persian. We emphasized the necessity of interventions to enhance the intercultural-competency of health personnel delivering health services at Afghan-concentrated communities in Iran.

### Limitations

Despite the resourceful findings of present study, there were some shortcomings that reduce the representativeness of results across different Afghan population in Iran. First, we conducted our study in urban areas where usually is the home for those refugees and immigrants with higher socioeconomic status and may not represent the underprivileged rural refugees and immigrants who may be of different sociocultural backgrounds and reproductive needs. Therefore, further studies among rural Afghan population is recommended. Second, we recruited the Afghan women at community health centers; therefore, a group of Afghan women whose access to such facilities is limited may have been missed. There were also some participants whose residential status appeared to be illegal and refuse to participate due to the fear of disclosure and potential arrest. Although, we tried to overcome this issue by engaging interviewers of Afghan origin and explaining the objective of this study for them; however, we might loss some valuable information on those who refused to participate. We also failed to collect the exact data on the gestational age at the first ANC.

## Conclusion

To the best of our knowledge, this study is the first study exploring the determinants and barriers towards adequate ANC among Afghan women in Iran. Almost a third of Afghan women in this study had adequate ANC (≥ 8 visits). Our study emphasized the important role of the personal knowledge and attitude toward ANC with adequate antenatal care among Afghan women in Iran. This could be addressed by well-oriented interventions and health education for Afghan women. It is recommended that future policies and interventions consider the specific demography and sociocultural concerns of Afghan people in Iran to improve the ANC access for expectant Afghan mothers. Conceiving an affordable health insurance with adequate coverage of maternity care services for Afghan refugees and immigrants should be a priority for central government. The collaboration between international agencies for refugees and immigrants support and national government should be emphasized to achieve an affordable health insurance for all refugees and immigrants in Iran. The efforts should be directed toward enhancing the social support, promoting the awareness and enhancing the reproductive health knowledge, and expanding the safety net services in order to increase the access and utilization of maternity cares for Afghan women in Iran.

## Supplementary information

**Additional file 1: Supplementary 1.** Questionnaire.

## Data Availability

Data could be available upon a reasonable request and with the permission of Tehran University of Medical Science ethical committee.

## Abbreviations

IRB: Institutional review board
ANC: Antenatal care
COR: Crude odds ratio
AOD: Adjusted odds ratio
TLS: Time-location sampling
PHI: Public health insurance
