# Supplementary material for: Barriers and associated factors for adequate antenatal care among Afghan women in Iran; findings from a community-based survey
Source: BMC Pregnancy Childbirth. 2020 Jul 28;20:427. doi: 10.1186/s12884-020-03121-5 (PMC7389441; doi:10.1186/s12884-020-03121-5)
Supplement: Supplementary file 1 — Additional file 1: Supplementary 1. Questionnaire. [file 12884_2020_3121_MOESM1_ESM.docx]

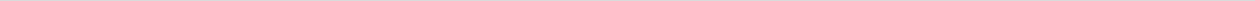


**Questionnaire for the study of prenatal care access among Afghan women in Iran**

Greetings,

This study is concerning the prenatal care service utilization and pregnancy outcome among Afghan women in Iran. You are kindly requested to participate in the study, which would definitely have importance in improving prenatal care services and health pregnancy for Afghan women in Iran. The interview will take not more than thirty minutes. The questions are related to your access and utilization of prenatal care services and the complications that you possibly experience during delivery. No information concerning you, as an individual will be passed to another individual or institution without your permission. Your participation is voluntary. If you agree to be included in the study, I would like to begin the interview. The study is approved by Kyoto University, Japan.

We would really appreciate your participation in this study.

Name of the Interviewer: …………………

Date of the Interview:……………………..

Place of Interview:…………………………

Address for Communication:

Omid Dadras

MD, MPH, DrPH candidate

Department of Health informatics

Graduate School of Medicine, Kyoto University.

Tel: Iran (+98911-405-8914) Japan (+8175-753-4350)

Email: [Omiddadras@yahoo.com](mailto:Omiddadras@yahoo.com)
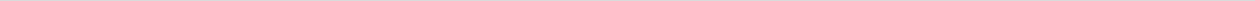


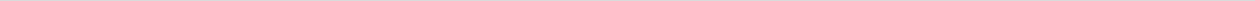


**Part I: General Information**

| **Q.No.** | **Questions** | **Category Coding** | **Skip** |
| --- | --- | --- | --- |
| **1.** | How old are you? | _______ years |  |
|  |  |  |  |
| **2.** | How long have you been | _______ years |  |
|  | living in Iran? |  |  |
| **3.** | What is your resident status | Holding a valid visa……….1 |  |
|  | In Iran? | Others ……………………..2 |  |
|  |  | *Please specify…..* |  |
| **4.** | Type of family you live | Nuclear ……………………1 |  |
|  | In | Joint………………………..2 |  |
|  |  | No. of Family members …… |  |
|  |  |  |  |
| **5.** | What is the highest level | Illiterate.……………………1 |  |
|  | of school you attended? | Primary…….. ….…………..2 |  |
|  |  | Secondary ...…..……………3 |  |
|  |  | High school ..……………....4 |  |
|  |  | College or higher……..…….5 |  |
| **6.** | How old were you when | _______years |  |
|  | you got married? |  |  |
| **7.** | How old is your | _______ years |  |
|  | husband ? |  |  |
| **8.** | Is your husband living | Yes ………………..…..….1 |  |
|  | with you? | No ………………….……..2 |  |
| **9.** | What is your husband | Afghan……………....…….1 |  |
|  | nationality | Iranian ……….…………....2 |  |
|  |  | Others ……………..………3 |  |
|  |  |  |  |
| **10.** | What is the highest level | Illiterate.……………………1 |  |
|  | of school your husband | Primary…….. ….…………..2 |  |
|  | attended? | Secondary ...…..……………3 |  |
|  |  | High school ..……………....4 |  |
|  |  | College or higher……..…….5 |  |
| **11.** | How many children do | _______ |  |
|  | you have? |  |  |
| **12.** | What is your present | Employed …………..…….1 |  |
|  | occupation? | Unemployed ...……………2 |  |
|  |  | Self-employed…………….3 |  |
|  |  | Others ……………………..4 |  |
|  |  |  |  |


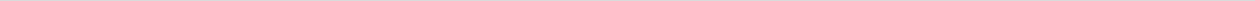


| **13.** | What is your husband | Employed ……………..….1 |  |
| --- | --- | --- | --- |
|  | present occupation? | Unemployed ...……………2 |  |
|  |  | Self-employed………….….3 |  |
|  |  | Others ……………………..4 |  |
|  |  |  |  |
| **14.** | How much is your | Rs.___________ |  |
|  | monthly family income? |  |  |
| **15.** | Which is the nearest | Govt Sub center / Primary |  |
|  | healthcare facility from | Health Center …………........1 |  |
|  | your house? | Govt Hospital………………..2 |  |
|  |  | Private clinic / hospital ……..3 |  |
|  |  | NGO………………………...4 |  |
| **16.** | How far is the nearest | ________ kilometers |  |
|  | healthcare facility from |  |  |
|  | your house? |  |  |
| **17.** | How do you travel to | Walk ………………………1 |  |
|  | this facility? | Public transport……………2 |  |
|  |  | Private vehicle..………..…..3 |  |
|  |  | Taxi ………………………..4 |  |
| **18.** | Do you own any kind of | Yes…………………………1 |  |
|  | health insurance? | NO …………………………2 |  |


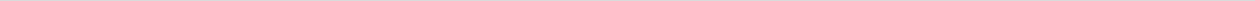


**Part II: Information on Maternal Healthcare Utilization**

**Fill up the following information from question 19 to question 40 with reference to the most recent pregnancy and delivery in the last year.**

| **Q.No** | **Questions** | | | | **Category Coding** | | | **Skip** |
| --- | --- | --- | --- | --- | --- | --- | --- | --- |
| **19.** | Did you register your pregnancy at the primary health center? | | | | Yes……………………………1 | | |  |
|  |  |  |  |  |  | | |  |
|  |  |  |  |  | No……………………….. …..0 | | |  |
| **20.** | Did you receive health check-up (PNC) during your last pregnancy at least once ? | | | | Yes……………………………1 | | | If ‘NO’ please go to question  number 30 |
|  |  |  |  |  |  | | |  |
|  |  |  |  |  | No……………………………..0 | | |  |
|  |  |  |  |  |  | | |  |
| **21**. | How many months pregnant were you when you first received antenatal care for this pregnancy? | | | | Months . . . . . . . . . . . . . .  Don't know . . . . . . . . . . . . . . . . . . . . . . 98 | | |  |
| **22.** | Why did you go for an PNC check-up? | | | | I was sick………………………………..…1 | | |  |
|  |  |  |  |  | Advised by a health worker ………..….......2 | | |  |
|  |  |  |  |  | Advised by my family members………...…3 | | |  |
|  | *(more than 1 answer can be* | | | | To start a regular check- up…......................4 | | |  |
|  | *marked)* | | | | Other reason, ………………………………5 | | |  |
|  |  | | | | *Please specify……* | | |  |
|  |  | | | |  | | |  |
| **23.** | Where did you go for your PNC check-ups? | | | | Govt. Sub-center / Primary Health Center ……..…......................................1 | | |  |
|  |  |  |  |  |  |  |  |  |
|  |  |  |  |  | Govt. Hospital……………....................2 | | |  |
|  |  |  |  |  | Private clinic / hospital ….……..….…..3 | | |  |
|  |  | | | | NGO…………………………..…….....4 | | |  |
|  |  | | | | Other place, ……..…………..…………5 | | |  |
|  |  | | | | *Please specify……* | | |  |
|  |  | | | |  | | |  |
| **24.** | Why did you choose the above health institution for checkup?  *(more than 1* *answer can be* *marked)* | | | Close to my house……………………..…1 | | | |  |
|  |  |  |  | Close to where I work ……………………2 | | | |  |
|  |  |  |  | Inexpensive………………….....................3 | | | |  |
|  |  |  |  | Behaviour of staff is good………………...4 | | | |  |
|  |  |  |  | Convenient timing………………………...5 | | | |  |
|  |  |  |  | Good quality service……………..…..........6 | | | |  |
|  |  |  |  | Others reason, ………………………….....7 | | | |  |
|  |  |  |  | *please specify…* | | | |  |
|  |  | | |  | | | |  |
| **25.** | Who examined  you during your  antenatal check-  ups? | | | Health personnel | | |  |  |
|  |  |  |  | Doctor …………………………….1 | | | |  |
|  |  |  |  | Midwife/Lady health visitor | | | |  |
|  |  |  |  | /ANM/Nurse……………………….2 | | | |  |
|  |  |  |  | Other health personnel ……............3 | | | |  |
|  |  |  |  | Non Health Personnel……….…………….4 | | | |  |
|  |  |  |  | *Please specify …….* | | | |  |
|  |  |  |  |  | | | |  |
| **26.** | How many times did you receive antenatal care during this pregnancy? | | | Number of times . . . . ….  Don't know . . . . . . . . . . . . . . . . . . . . . . 98 | | | |  |
| **27.** | Did you receive  the following services, at-least once, during your  pregnancy check-ups ? | | |  | |  |  | |
|  |  |  |  |  | |  |  | |
|  |  |  |  | **Yes** | | **No** | **Don’t know** | |
|  |  |  |  | **1** | | **0** | **98** | |
|  |  |  |  |  | |  |  | |
|  |  |  |  |  | |  |  | |
|  | **27.1** | Tetanus | |  | |  |  | |
|  | Injection on your | | |  | |  |  | |
|  | arm | |  |  | |  |  | |
|  | **27.2** Iron tab/syrup | | |  | |  |  | |
|  | folic acid tab | | |  | |  |  | |
|  | **27.3** | Your weight | |  | |  |  | |
|  | was checked | | |  | |  |  | |
|  | **27.4** | Blood/ Urine | |  | |  |  | |
|  | Tests conducted | | |  | |  |  | |
|  | **27.5** | Blood | |  | |  |  | |
|  | pressure examined | | |  | |  |  | |

|  | **28.** | During your  pregnancy, did you receive advice on : | | | |  |  |  | |
| --- | --- | --- | --- | --- | --- | --- | --- | --- | --- |
|  |  |  |  |  |  | **Yes** | **No** | **Don’t know** | |
|  |  |  |  |  |  | **1** | **0** | **98** | |
|  |  | **28.1** | Complications / | | |  |  |  | |
|  |  | problems during | | | |  |  |  | |
|  |  | pregnancy | | | |  |  |  | |
|  |  | **28.2** | | Place | of |  |  |  | |
|  |  | delivery | | |  |  |  |  | |
|  |  | **28.3** | | Nutrition | |  |  |  | |
|  |  | during | | pregnancy | |  |  |  | |
|  |  | **28.5** Baby care | | | |  |  |  | |
|  |  |  | | | |  |  |  | |
|  | **29.** | Why didn’t you | | | | I was healthy ……………………………….1 | | |  |
|  |  | attend | | health | | I thought it was unnecessary….....................2 | | |  |
|  |  | check-up (ANC) | | | | Expenses of Check-up was | | |  |
|  |  | during | |  |  | unaffordable………………………………..3 | | |  |
|  |  | pregnancy? | | |  | Clinic is too far away from home…………..4 | | |  |
|  |  |  | |  |  | Family members disapproved ……………..5 | | |  |
|  |  | *(can mark* *multiple answers)* | | | | Poor transportation facility to the health | | |  |
|  |  |  |  |  |  | facility ………………….………… ……….6 | | |  |
|  |  |  | | |  | I was scared of being discriminated or being poorly treated. …………………………………….. 7 | | |  |
|  |  |  | |  |  | No one to accompany me ………………….8 | | |  |
|  |  |  | |  |  | I believed the services were no good……….9 | | |  |
|  |  |  | |  |  | Did not know where to go…………………10 | | |  |
|  |  |  | |  |  | No female provider was available………….11 | | |  |
|  |  |  | |  |  | The service hour was inconvenient………...12 | | |  |
|  |  |  | |  |  | Long waiting time………………………….13 | | |  |
|  |  |  | |  |  | Religious and cultural reasons……………..14 | | |  |
|  |  |  | |  |  | Better service at home……………………...15 | | |  |
|  |  |  | |  |  | Other reason…………………..…………….16 | | |  |
|  |  |  | |  |  | *please specify……..…* | | |  |
|  |  |  | |  |  |  | | |  |
|  |  |  | |  |  |  | | |  |
